# A Perspective of the Epidemiology of Rabies in South Africa, 1998–2019

**DOI:** 10.3390/tropicalmed9060122

**Published:** 2024-05-22

**Authors:** Ayla J. Malan, Andre Coetzer, Cayla Bosch, Nicolette Wright, Louis H. Nel

**Affiliations:** 1 Department of Biochemistry, Genetics and Microbiology, Faculty of Natural and Agricultural Sciences, University of Pretoria, Pretoria 0002, South Africa; 2 Global Alliance for Rabies Control, Manhattan, KS 66502, USA

**Keywords:** rabies burden, epidemiology, South Africa, spatio-temporal analysis, surveillance

## Abstract

Despite the implementation of various control strategies aimed at eliminating canine-mediated rabies, the disease is still endemic in up to 150 countries across the world. Rabies remains endemic to South Africa, with various reservoir species (both wildlife species and domestic dogs) capable of maintaining rabies infection, and the epidemiology of the disease is yet to be adequately defined. As such, this study used surveillance data collected between 1998 and 2019 from the two diagnostic laboratories in the country for a statistical space–time analysis to determine regions where significant disease clusters could occur. In addition, the robustness of surveillance activities across the country was evaluated through the mathematical evaluation and visualization of testing rates based on the average number of samples tested per species group. In our study, various significant disease clusters were detected for domestic animals, wildlife and livestock. The significant disease clusters for domestic animals and livestock were primarily restricted to eastern South Africa, while the significant disease clusters in wildlife species were detected across northern and western South Africa. Furthermore, the testing rates identified districts from various provinces where surveillance activities could be considered inadequate, consequently influencing the geographical range of the observed clusters. These results could be used to direct intervention campaigns towards high-risk areas, while also allocating the required resources to improve surveillance in the surrounding areas where surveillance was deemed inadequate.

## 1. Introduction

Anecdotal evidence suggests that canine-mediated rabies has been known to man since ancient times [1]. Despite being a vaccine-preventable disease [2] and recent efforts to eliminate the disease through mass dog vaccination [3,4,5], canine-mediated rabies still causes tens of thousands of human deaths annually, with most of these deaths occurring in Africa and Asia. In Africa, where canine-mediated human rabies is estimated to result in the death of 21,000 people every year, disease persistence is attributed to the cycle of neglect, which is perpetuated by limited surveillance and disease burden data [6,7]. As a result, rabies elimination is not prioritized beyond the political realm, resulting in the ongoing occurrence of rabies cases in animals and humans alike [3,8,9].

South Africa—a country situated at the southernmost tip of the African continent—is divided into nine administrative provinces, viz. the Limpopo (LP), North West (NW), Northern Cape (NC), Eastern Cape (EC), KwaZulu-Natal (KZN), Free State (FS), Western Cape (WC), Mpumalanga (MP), and Gauteng (GP) provinces. In addition, the country shares political borders with Namibia, Botswana, Zimbabwe, Mozambique, Lesotho and Eswatini (previously the Kingdom of Swaziland). Anecdotal evidence suggests that canine rabies was first established in South Africa during the early 1900s [10], and today six of the nine South African provinces (viz. LP, NW, MP, KZN, EC, and FS provinces) remain endemic for canine-mediated rabies. In contrast, the remaining provinces (NC, WC, and GP provinces) only experience sporadic canine-mediated rabies outbreaks and could be considered vulnerable to outbreaks but not endemic [11,12,13,14,15,16].

The dissemination of rabies in South Africa is further complicated by various sylvatic species that can maintain and transmit rabies. More specifically, three sylvatic reservoir species (predominantly black-backed jackals (*Canis mesomelas*), bat-eared foxes [*Otocyon megalotis*] and aardwolf [*Proteles cristatus*]) have been suggested to be capable of maintaining the canine variant of the *Lyssavirus rabies* (RABV) in South Africa [11,17,18,19,20,21,22,23,24]. In addition to the sylvatic species capable of maintaining the canine variant of RABV, various members of the *Herpestidae* family can maintain the mongoose variant of RABV that is phylogenetically distinct from the canine variant of RABV [20,21,22,23]. Furthermore, various rabies-related lyssaviruses have also been detected in South Africa. These include *Lyssavirus duvenhage* (DUVV), *Lyssavirus lagos* (LBV), *Lyssavirus caucasicus* (WCBV), Matlo bat lyssavirus (MBLV, pending recognition by the International Committee on Taxonomy of Viruses (ICTV)), Phala bat lyssavirus (PBLV, pending ICTV recognition), as well as *Lyssavirus mokola* (MOKV). With the exception of the latter, MOKV, all of these are known to infect various bat species [25,26]. Although MOKV has been isolated from domestic cats in South Africa, the reservoir host remains unknown [27]. The public health impact of these rabies-related viruses is considered negligible in comparison to the burden of canine-mediated human rabies [23,28,29].

South Africa has made progress towards freedom from canine-mediated human rabies through dog vaccination campaigns across the country [30] and the development of a national strategic plan [31]. However, it is evident that strategic disease interventions will be required to eliminate the disease in a resource-considerate manner. To this end, an in-depth understanding of the epidemiology of rabies is required so that high-risk areas can be identified and targeted. It is thus imperative that existing surveillance data be scrutinized and supplemented if the epidemiology of rabies in South Africa is to be fully elucidated.

Although molecular epidemiological analyses had been extensively used to supplement the existing surveillance data [11,12,14,15,16,18,19,32,33,34,35,36,37,38,39,40], the studies were primarily limited to inferring viral relatedness between geographic localities and species. As a result, those investigations were not necessarily of major significance in pinpointing specific high-risk areas that should be targeted during astute strategic intervention campaigns. In addition to the various molecular epidemiological analyses undertaken to date, two previous studies used rabies surveillance records from South Africa to gain an improved understanding of the epidemiology of rabies in South Africa. The first of these studies undertook temporal distribution and spatial analyses for rabies in different species groups from 1993 to 2005 using rabies case data reported to the National Department of Agriculture, Land Reform and Rural Development (DALRRD) [41]. This study identified various rabies enzootic regions throughout the country for various species groups, viz. domestic dogs were enzootic throughout the entire KZN, rabies outbreaks in jackals were mainly restricted to the LP province, rabies cases in bat-eared foxes spread across the NC, western NW, western FS, northwest EC, and northern WC provinces, and rabies in mongoose species had spread from the central plateau (FS) into six neighboring provinces (the NW, GP, MP, NC, EC and WC provinces). Furthermore, the researchers found that almost 80% of all rabies cases in the country were from domestic dogs and production animals. Despite providing valuable insight into the distribution of rabies cases across South Africa, this study was limited to using the surveillance data that the state veterinarians had reported to DALRRD, where data was quality-controlled and validated with data provided by the two rabies diagnostic laboratories in South Africa. As a result, the investigation could not use the combined animal rabies case data of the two laboratories, which Gummow et al. believed would have been more accurate [41]. Similar to the previous investigation, the second study also used the surveillance data that had been reported to the national database and maintained by DALRRD for their investigation. In this investigation, however, the authors used the surveillance data that had been collected between 1993 and 2019 as part of a spatio-temporal analysis focusing primarily on the role of wildlife in the spread of rabies in the country [42]. This study also found that domestic dogs and livestock constituted most of the reported rabies cases in the country, with most of the significant disease clusters restricted to the eastern half of the country. Of the 13 significant disease clusters detected, wildlife species were encountered in nine of the clusters spread mostly across the north and eastern provinces in the country. Both studies confirmed that most canine rabies-positive cases originated from the KZN province, jackal rabies was restricted to the northern regions of the LP and NW, and bat-eared fox rabies cases were mainly restricted to the western half of the country [41,42].

Although epidemiological investigations for rabies in South Africa had been undertaken prior to this investigation, none of the investigations had, to the best of our knowledge, relied on a comprehensive dataset of surveillance data generated by both rabies diagnostic laboratories. Furthermore, none of the past investigations had taken the robustness of the surveillance data into consideration when inferring disease prevalence throughout the country. To this end, our study aimed to use both the positive and negative rabies case data for all recorded animal rabies cases in the country for a statistical space–time analysis to determine regions where significant disease clusters could be seen. Additionally, the robustness of rabies surveillance activities was evaluated throughout the country by determining the rabies testing rates for different animal groups. This approach enabled us to not only identify significant disease clusters but also determine geographic localities where surveillance might be limited.

## 2. Materials and Methods

### 2.1. Data Collection and Curation

Rabies surveillance data collected between 1998 and 2019 were obtained from the two laboratories responsible for rabies diagnosis in South Africa using the fluorescent antibody test (FAT). These laboratories were the Central Veterinary Laboratory (CVL) situated in the GP province (Agricultural Research Council–Onderstepoort Veterinary Research (ARC-OVR), Rabies Unit) and the Provincial Veterinary Laboratory (PVL) in the KZN province (Allerton Provincial Veterinary Laboratory). These two rabies diagnostic laboratories are the only accredited laboratories that can receive and confirm suspected animal rabies cases in South Africa. Suspected human rabies cases are sent to the National Institute for Communicable Diseases (NICD) for diagnostic confirmation. The surveillance data—consisting of the (i) year of diagnosis, (ii) species subjected to diagnosis, (iii) location of sampling (if known), and (iv) diagnostic outcome—was used for subsequent analyses. All the data were combined and consolidated into a single Excel spreadsheet (Microsoft Office, 2016), after which the species in the dataset were categorized as follows: domestic animals (domestic dogs and cats), livestock (bovine, caprine, equine, porcine and ovine species), mongoose (all mongoose species), and sylvatic species (all wildlife species excluding mongoose species). Rabies-related cases detected in bat species were excluded from the analysis as bat species in South Africa are not known to transmit RABV and no evidence exists that species cross-over events between canines and bats occur [26]. Once the data had been categorized, a descriptive analysis was conducted for all data originating from across South Africa from 1998 to 2019 in Excel (Microsoft Office, 2016).

### 2.2. Spatio-Temporal Analysis of Rabies Cases in South Africa

The SaTScan™ software (version 10.0.2) was used for a spatio-temporal cluster analysis using a discrete Bernoulli space–time probability model, and 99,999 Monte Carlo replicates for each species group (i.e., domestic animals, livestock, wildlife, and mongoose species) [43]. Of all records made available to this investigation (*n* = 37,039), those with unknown geographical location data (*n* = 2091) were excluded from the spatio-temporal analysis. The Bernoulli model was used to determine geographically defined disease clusters based on the number of cases and controls encountered within a particular region (in this case, all positive and negative rabies cases) [43]. Significant clusters were defined as those with a *p*-value smaller than 0.05 (*p* < 0.05), and all clusters with a *p*-value of >0.05 were excluded. Furthermore, a relative risk—defined as the estimated risk of having a case within the cluster divided by the estimated risk of encountering a case outside the cluster—was also calculated for each cluster. All maps relating to the SaTScan™ outputs were created using QGIS Desktop (version 3.30.0).

### 2.3. Calculating Rabies Testing Rates for South Africa

Using an approach described elsewhere [44] and the 2022 human census data [45], the ‘all-animal-per-human testing rate’ (AAHR) (Equation (1)), the ‘domestic-animal-per-human testing rate’ (DAHR) (Equation (2)), and the ‘wildlife-per-human testing rate’ (WHR) (Equation (3)) was calculated for each district in South Africa using the formulas provided below. To account for any year-to-year variation, while providing a fair overview of the contemporary passive surveillance capacity, the testing rates provided here were based on the last 10 years of surveillance data (2010–2019) and the most recent human population estimates released in 2022.

(1) $$\mathrm{AAHR}=\frac{\mathrm{Average}\;\mathrm{number}\;\mathrm{of}\;\mathrm{all}\;\mathrm{animals}\;\mathrm{tested}/\mathrm{year}}{\mathrm{Estimated}\;\mathrm{human}\;\mathrm{population}}\times 100,000$$

(2) $$\mathrm{DAHR}=\frac{\mathrm{Average}\;\mathrm{number}\;\mathrm{of}\;\mathrm{domestic}\;\mathrm{animals}\;\mathrm{tested}/\mathrm{year}}{\mathrm{Estimated}\;\mathrm{human}\;\mathrm{population}}\times 100,000$$

(3) $$\mathrm{WHR}=\frac{\mathrm{Average}\;\mathrm{number}\;\mathrm{of}\;\mathrm{wildlife}\;\mathrm{animals}\;\mathrm{tested}/\mathrm{year}}{\mathrm{Estimated}\;\mathrm{human}\;\mathrm{population}}\times 100,000$$

In addition to each testing rate described above, defined threshold values—determined by Minhaj et al.—were used to determine the adequacy of each of the different testing rates (AAHR: 1.9; DAHR: 0.8; WHR: no threshold value defined) [41]. More specifically, testing rate values above the threshold value indicate districts where surveillance and rabies testing were considered to be adequate, while districts below the threshold value had limited surveillance and rabies testing.

### 2.4. Dog Population Estimates

The estimated domestic dog densities for each district in the country was calculated using dog: human ratios. The dog population estimates were calculated at local municipality level as this provided greater resolution to the data and allowed for each local municipality to be classified as either “urban”, “peri-urban”, or “rural” according to the South African Municipal Infrastructure Investment Framework (MIIF) classification system [45]. As various studies from different provinces in the country had found varying ratios for urban and rural locations, the published ratios were used to calculate average dog: human ratios for urban and rural locations used in this investigation [15,42,46,47]. The peri-urban dog: the human ratio was determined by calculating the mean ratio between the urban and rural ratios. Therefore, the estimated dog populations in urban areas were calculated using a ratio of 1:23 (dog: human), a ratio of 1:16.7 (dog: human) for peri-urban regions and a ratio of 1:10.5 (dog: human) for rural areas. Using the estimated dog population size at a local municipality level, the dog population for each district was calculated (the sum of the estimated dog populations in all local municipalities), and the estimated dog density per km^2^ was calculated by dividing the estimated dog population by the total area for the district [45].

Additionally, it was previously shown that geographical areas with a domestic dog density as low as 1.36 dogs/km^2^ could maintain rabies transmission in local dog populations [48]. As such, the conservative cut-off value for domestic dog populations capable of maintaining rabies transmission in this investigation was also set to 1.36 dogs/km^2^.

## 3. Results

From the total of 37,039 suspected rabies samples submitted for diagnostic confirmation between 1998 and 2019, 11,907 (32.1%) tested positive for rabies (Table S1). Of the 18,936 suspected domestic dog samples submitted, 6682 (35%) tested positive (Table S1). Livestock had the second-highest number of samples tested for rabies (*n* = 6341 tested, 47.7% positive), followed by wildlife (*n* = 4577 tested, 24.9% positive), mongoose species (*n* = 3790 tested, 29.2% positive), and domestic cats (*n* = 3395 tested, 9.8% positive) (Table S1).

**Figure 1:**
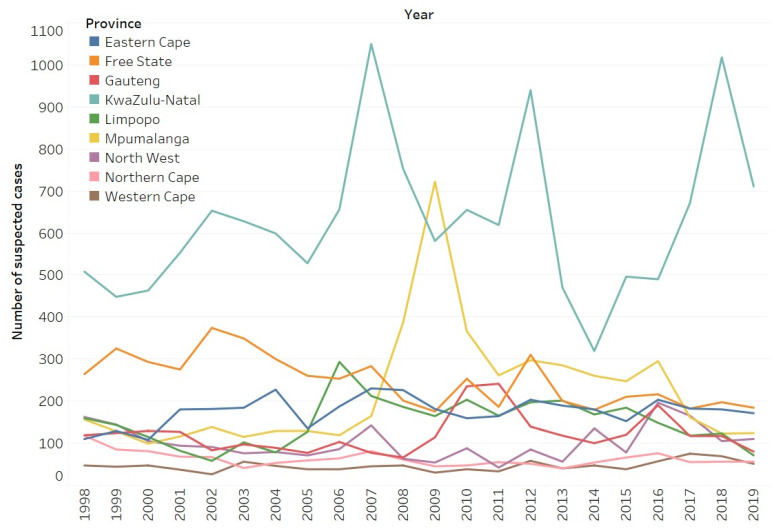
Number of samples submitted for diagnostic confirmation per province across South Africa between 1998 and 2019.

While the average number of samples subjected to diagnostic confirmation remained fairly standard across the years (min: 1418, max: 2279, and mean: 1738), not all of the provinces contributed equally in terms of submitting samples for diagnosis. For example, between 1998 and 2019, most of the samples sent for diagnostic confirmation originated from the KZN province (average of 621 suspect cases per year), with the exception of one year (2009) when most samples originated from the MP province (Figure 1, Table S2). During the same time period, the NC province contributed the fewest number of suspect rabies cases for diagnosis (an average of 60 suspect cases per year) (Figure 1, Table S2).

**Figure 2:**
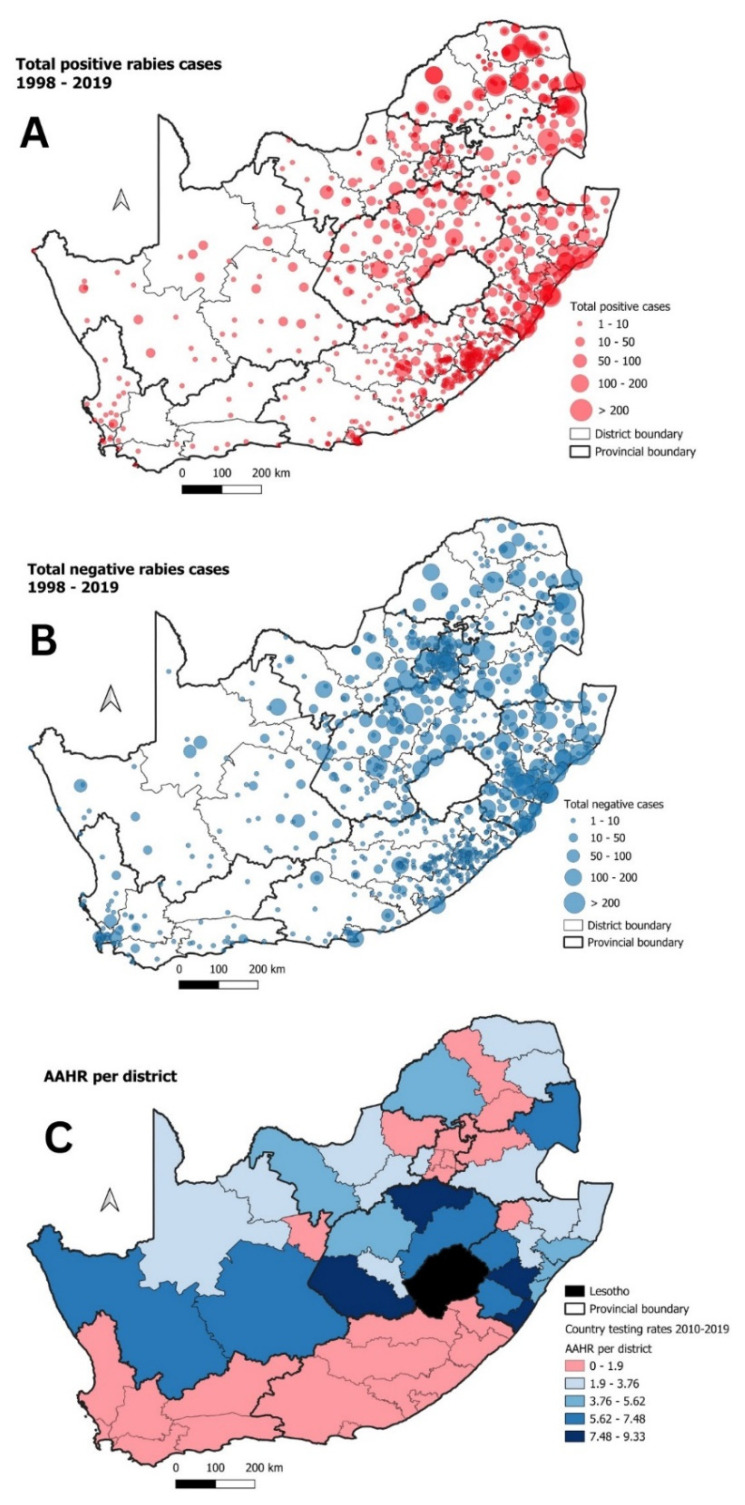
(**A**) The total positive rabies cases in South Africa between 1998 and 2019; (**B**) total negative rabies cases in South Africa between 1998 and 2019; and (**C**) AAHR values per district across South Africa (2010–2019) where districts below the threshold value are indicated in red.

From the surveillance dataset (1998 to 2019), the majority of both rabies-positive and rabies-negative samples originated from the eastern half of the country (Figure 2A,B). To determine whether animal health professionals operating in geographical areas with few or no rabies cases were undertaking adequate surveillance, the AAHR across South Africa was evaluated (Figure 2C). The AAHR for all the districts in the FS and NC provinces were consistently higher than the defined threshold value of 1.9 (Figure 2C). In the EC, the AAHR for six of the eight districts fell below the defined threshold value of 1.9. The remaining provinces (WC, GP, MP, LP, KZN, and NW) all had at least one district for which the AAHR was lower than the threshold value of 1.9.

### 3.1. Domestic Animals

**Figure 3:**
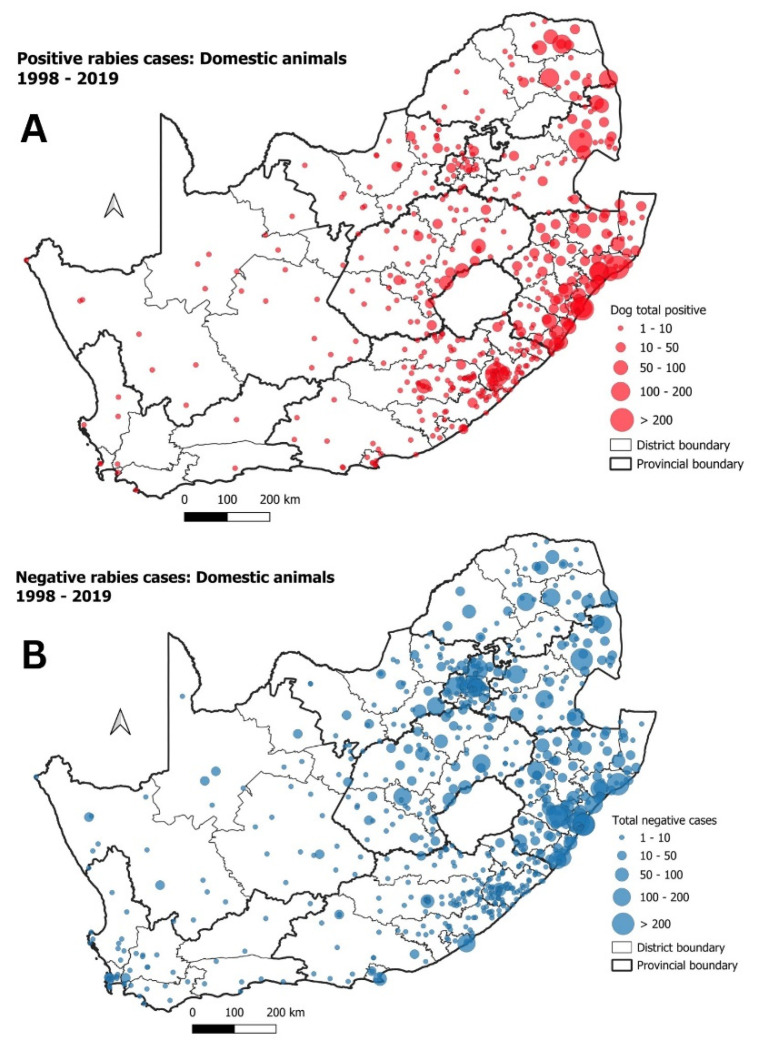
(**A**) Positive rabies cases in domestic animals in South Africa between 1998 and 2019; (**B**) negative rabies cases in domestic animals during the same period.

Of all suspect rabies cases, domestic dogs and domestic cats accounted for 7014 of all positive rabies cases (59%) between 1998 and 2019. Most positive rabies cases were distributed across eastern South Africa, with the highest numbers of canine rabies cases originating from the KZN province (Figure 3A,B).

**Figure 4:**
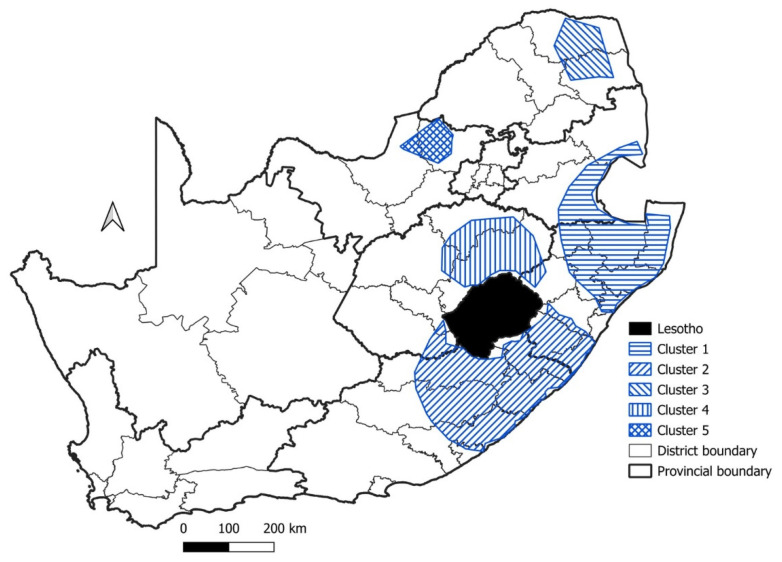
Significant disease clusters (indicated by the blue hashed lines) for domestic animals in South Africa, 1998–2019.

Using the surveillance data from domestic animals in the spatio-temporal cluster analysis, five significant disease clusters were detected for domestic animals (Figure 4, Table 1). The first significant disease cluster (Cluster 1) was located along the eastern seaboard of KZN where it encompassed the country of Eswatini, while Cluster 2 spanned across the KZN and EC provinces (Figure 4). These two clusters had high numbers of observed rabies cases (1455 and 935, respectively), far exceeding the numbers of expected rabies cases. The two smallest clusters (Cluster 3 and 5) were the most contemporary, with both clusters being detected after 2010 in northern SA (Figure 4, Table 1). The final significant disease cluster (Cluster 4) was mainly restricted to the FS province, bordering the country of Lesotho.

**Figure 5:**
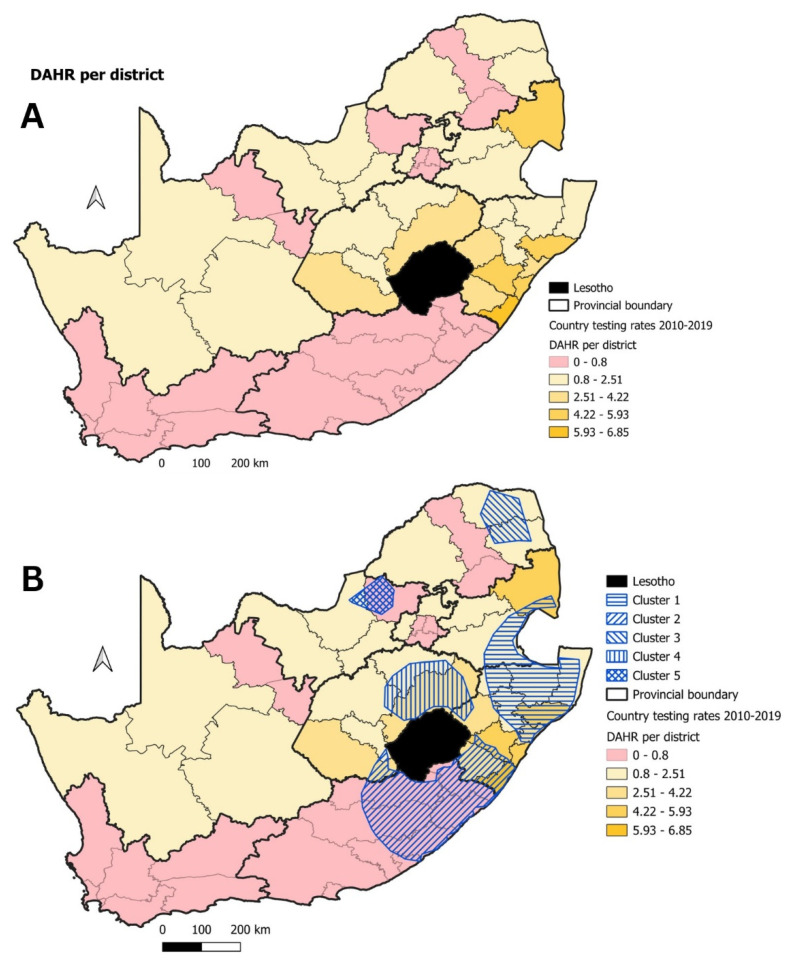
(**A**) DAHR testing rates for districts across South Africa (2010–2019) with districts below the defined threshold value indicated in red; (**B**) significant disease clusters (indicated by the blue hashed lines) for domestic animals with the DAHR values.

To determine whether geographical areas without any significant domestic animal rabies disease clusters were due to inadequate surveillance, the DAHR was calculated and visualized for each district in the country (Figure 5A). Across South Africa, 21 of the 52 districts (40%) had a DAHR testing rate below the required threshold. More specifically, all the districts in three provinces (FS, KZN, and MP) had DAHR values above the required threshold. By comparing the spatio-temporal cluster analysis output for domestic animals with the DAHR testing rate, Clusters 1, 3, and 4 geographically coincided with districts where the DAHR values were higher than the threshold (Figure 5B). Cluster 2 and 5, however, largely encompassed districts in the EC and NW provinces where the DAHR values were below the defined threshold value of 0.8 (Figure 5B).

**Figure 6:**
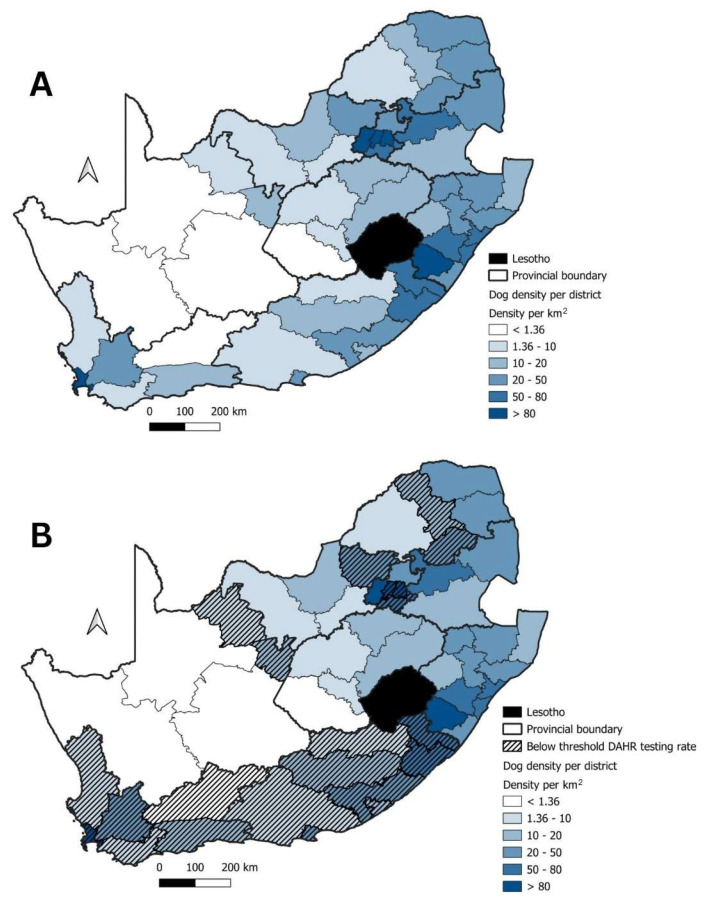
(**A**) the domestic dog densities in km^2^ per district across South Africa, districts below the threshold value are shown in white; (**B**) the below-threshold DAHR testing rate values (indicated using black hashed lines) in relation to the domestic dog density is indicated as shading on the map.

To identify areas where dog population densities were theoretically too low to maintain rabies, and thus negate the need for DAHR values above the required threshold—the estimated domestic dog densities per district was determined. Based on the spatio-temporal cluster analysis, all five significant disease clusters were detected across districts where the estimated domestic dog density exceeded the minimum value of 1.36 dogs/km^2^ (Figure 6A). Of the 21 districts were the DAHR testing rate was below the required threshold, the estimated dog population density was theoretically too low to maintain rabies in only one district located in the WC province (Figure 6A,B). In the remaining 20 districts, the estimated dog population density was deemed high enough to theoretically sustain rabies transmission.

### 3.2. Mongooses and Other Wildlife species

**Figure 7:**
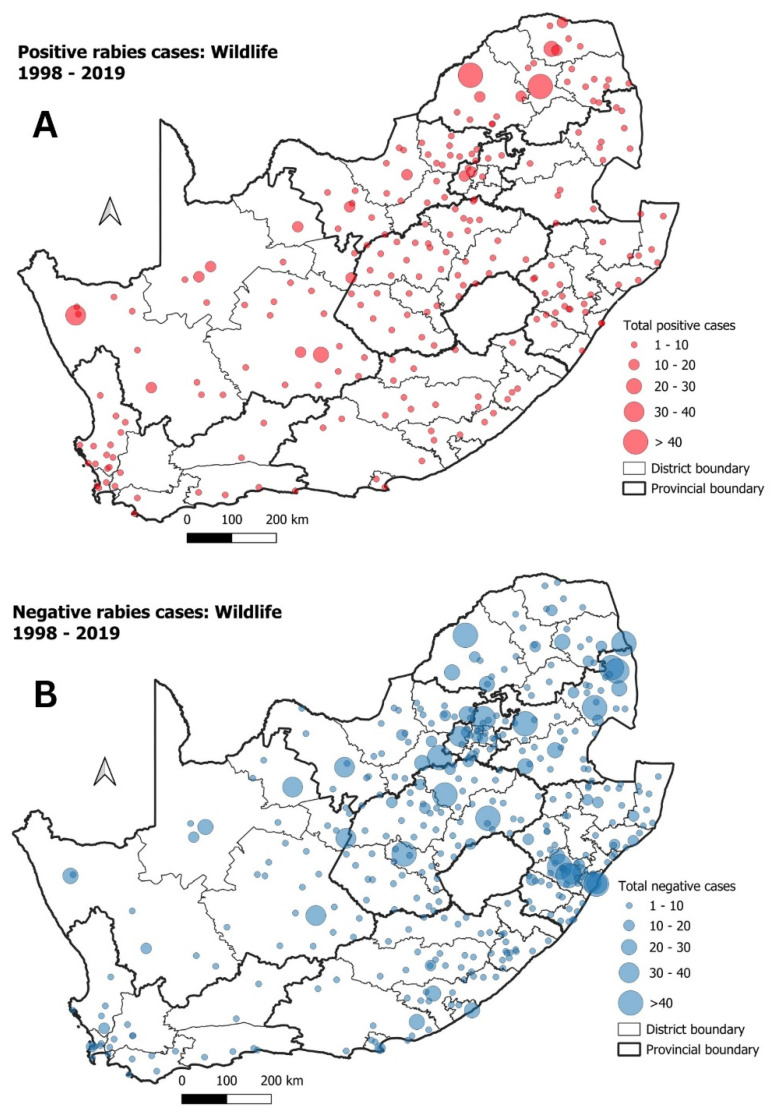
(**A**) Positive rabies cases in wildlife species (excluding mongoose species) in South Africa between 1998 and 2019; (**B**) negative rabies cases in wildlife species (excluding mongoose) during the same period.

Wildlife species (excluding mongoose species and bats) accounted for 1141 (10%) of all positive rabies cases in the country between 1998 and 2019 (Table S1). Positive rabies cases for various wildlife species were distributed throughout the country, with the highest numbers being recorded in the LP province and the western part of the NC province (Figure 7A). The negative rabies cases were similarly dispersed throughout the country, with the highest number of samples originating from the central and northern districts in the country (MP, GP, NW, FS and western LP) (Figure 7B).

**Figure 8:**
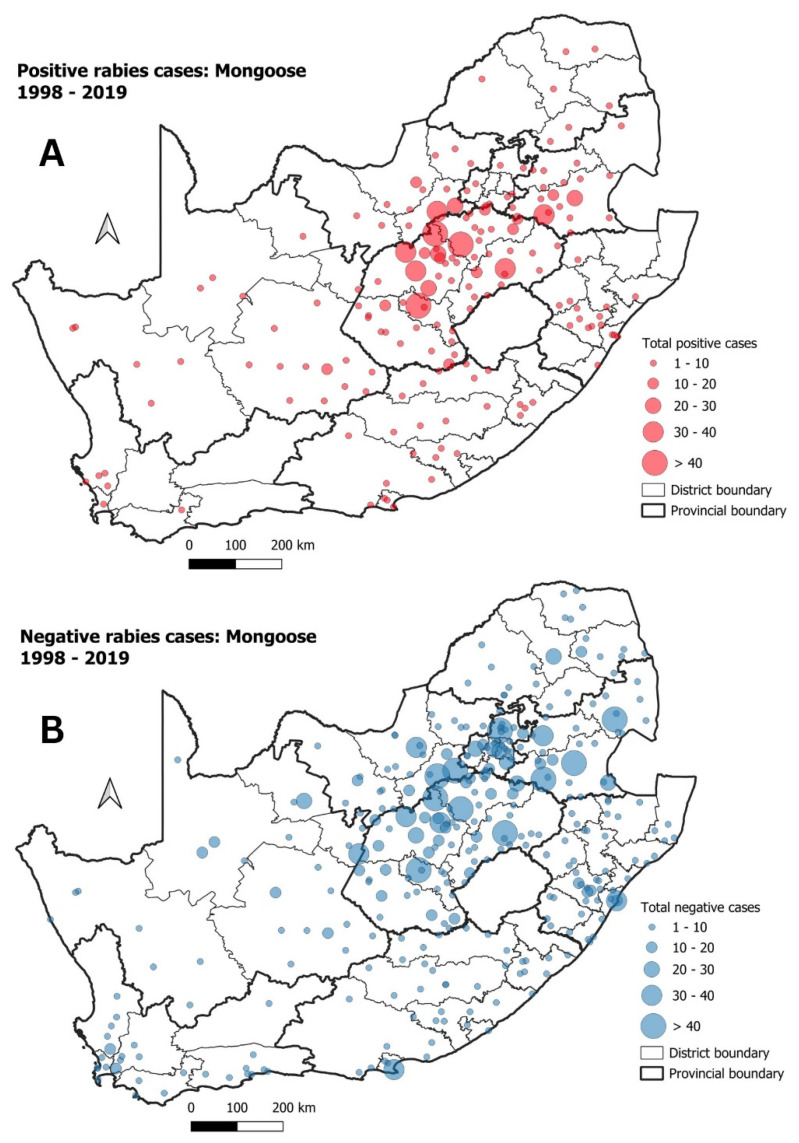
(**A**) Positive rabies cases in mongoose species in South Africa between 1998 and 2019; (**B**) negative rabies cases in mongoose species during the same period.

Rabies cases in mongoose species produced the lowest number of positive rabies cases during our study period, with only 1107 (9%) of all suspected rabies cases originating from mongooses testing positive (Table S1). The highest numbers of positive cases were reported from the central plateau of the country (Figure 8A), with the number of reported negative rabies cases following a similar trend (Figure 8B).

**Figure 9:**
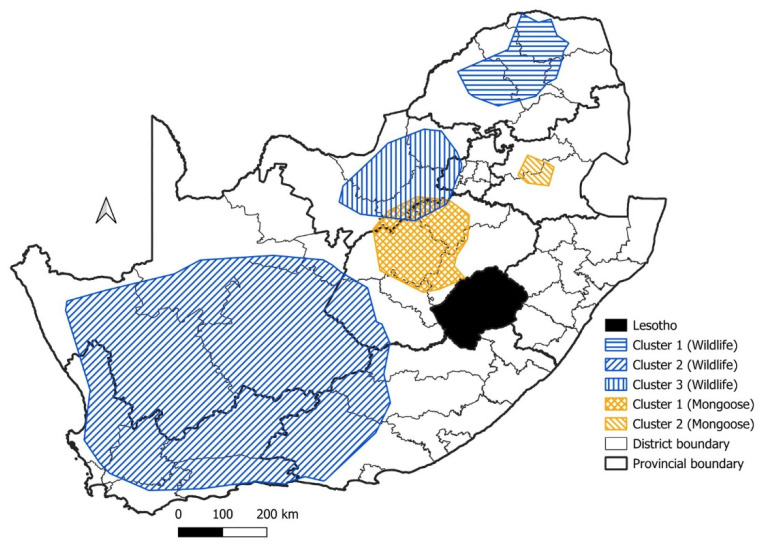
Significant disease clusters for wildlife species (indicated by the blue hashed lines) and mongoose species (indicated by the orange hashed lines) across South Africa, 1998–2019.

Applying the same methodology as described above, three significant disease clusters were detected for wildlife species (excluding mongooses) (Figure 9). Two of the clusters (Cluster 1 and 2) were detected between 1998 and 2008, while the last remaining cluster (Cluster 3) was active between 2016 and 2019 (up to the end of the study period) (Table 2). Cluster 1 was restricted to the central part of the LP province and overlapped to some extent with the significant disease cluster in domestic animals discussed earlier (Figure 4). While Cluster 3 covered large parts of the NW province, it also expanded into the western edges of the GP province and the northern edges of the FS province. This cluster also overlapped to some extent with the significant disease cluster in domestic animals discussed earlier (Figure 4). The final, and largest cluster (Cluster 2), covered a vast geographical area that extended into four provinces (NC, WC, EC and the FS province) (Figure 9). In addition, two separate significant disease clusters could be observed for mongoose species (Figure 9). The first cluster (Cluster 1: Mongoose) was restricted to the FS province with the highest number of observed cases between 1998 and 2008 (Table 2). The second cluster (Cluster 2: Mongoose) occupied a smaller geographic area in the MP province and had self-terminated by the end of 2005 (Figure 9; Table 2).

**Figure 10:**
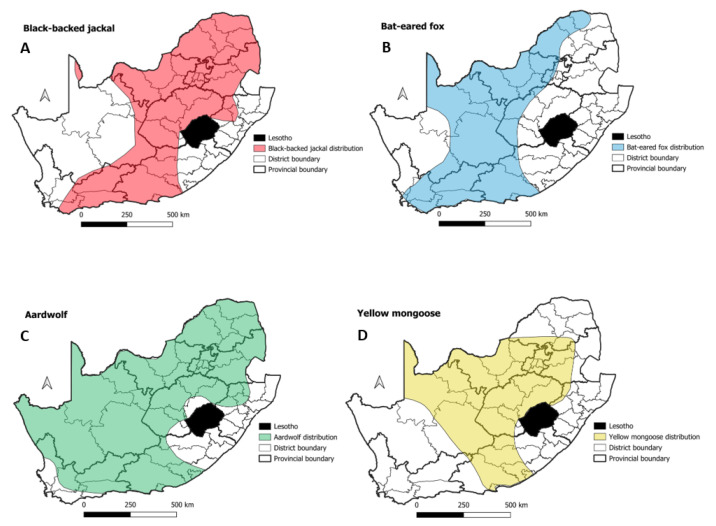
Species distributions for black-backed jackals (**A**), bat-eared foxes (**B**), aardwolf (**C**), and yellow mongoose species (**D**). Species distributions adapted using data from the EWT (https://ewt.org.za/red-list/ (accessed on 22 February 2024)).

**Figure 11:**
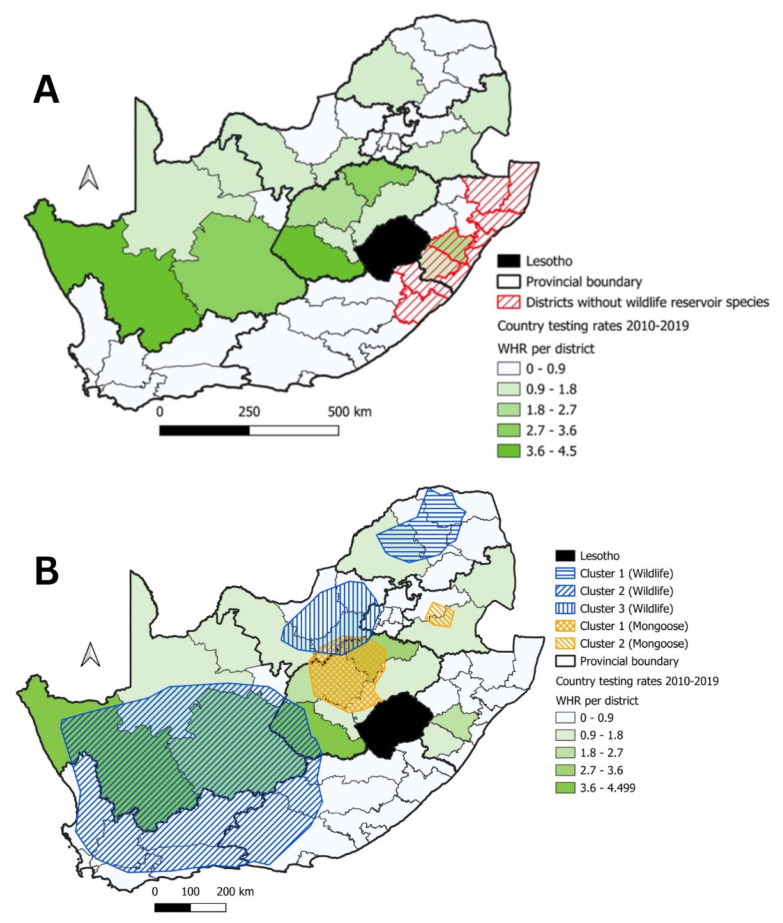
(**A**) WHR testing rate values (2010–2019) for each district indicated in green shading with districts that do not have wildlife reservoir species indicated in red hashed lines; (**B**) significant clusters for wildlife (indicated in blue hashed lines) and mongoose species (indicated in orange hashed lines) indicated in relation to the WHR.

Following on from the identification of the significant disease cluster in mongooses and other wildlife species, the species distributions of the country’s sylvatic reservoir species were used to define which districts in the country had the presence of one or more of the sylvatic species that could maintain rabies transmission. This approach found that 42 of the 52 (81%) districts in South Africa formed part of the home range of one or more of sylvatic reservoir species (Figure 10). The remaining 10 districts that did not overlap with the home range of one or more sylvatic species were located in the KZN and EC provinces (Figure 10) Thereafter, the WHR values were calculated using the surveillance data collected over the course of the last 10 years (2010–2019). Although no threshold value had been defined for the WHR, it still provided a useful indication of the wildlife surveillance activities within the home ranges of the sylvatic species known to maintain rabies transmission in South Africa (Figure 11). More specifically, the highest WHR values were observed in districts from the FS and NC provinces, while the WHR was lower in at least one district in each of the remaining provinces of the country (Figure 11A). By comparing the locations of the significant disease clusters in wildlife species and mongooses with the WHR, all the significant disease clusters included districts with WHR values that were both low and high (Figure 11B).

### 3.3. Livestock

**Figure 12:**
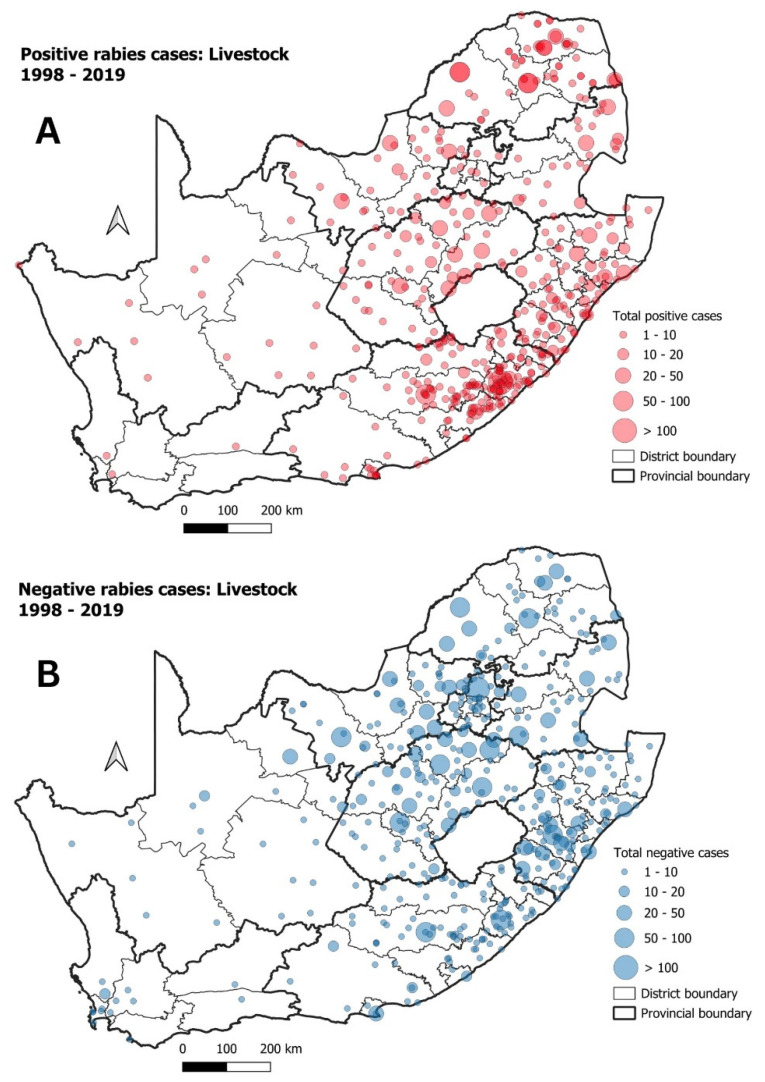
(**A**) Positive rabies cases in livestock in South Africa between 1998 and 2019; (**B**) negative rabies cases in livestock during the same period.

Livestock had the second-highest number of suspected rabies cases, with 2645 of the total cases (22%) testing positive for rabies (Table S1). As was observed for domestic animals, most of the positive and negative rabies cases for livestock were reported from the eastern half of the country (Figure 12A,B). The highest number of positive rabies cases originated from the EC province (Figure 12A), followed by the KZN, FS and NW provinces. In contrast to the high number of positive cases, relatively few negative cases originated from the EC province (Figure 12B), with higher numbers of negative cases originating from the KZN, FS and GP provinces.

**Figure 13:**
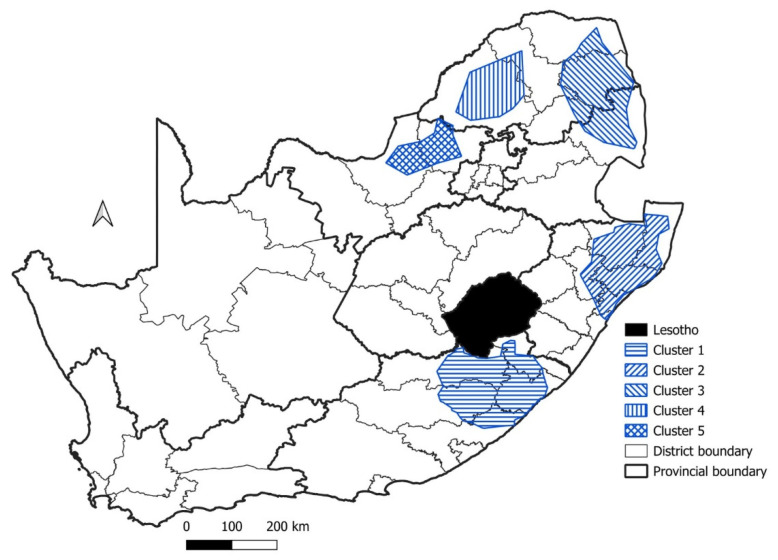
Significant disease clusters for livestock in South Africa between 1998 and 2019.

For rabies in livestock species, five separate significant disease clusters were detected (Figure 13). Four of the five significant disease clusters were restricted to provincial boundaries, viz. EC (Cluster 1), KZN (Cluster 2), LP (Cluster 4) and NW (Cluster 5) provinces. In contrast, one significant disease cluster (Cluster 3) ranged across the MP and LP provinces (Figure 13). The most contemporary of these clusters (Cluster 5) was only detected between 2014 and 2015, with only 44 observed rabies cases (Table 3). Two of the clusters (Cluster 2 and 4) were detected prior to 2009 with varying cluster durations. Cluster 1 was by far the most severe with 410 observed cases between 2002 and 2012 (Table 3).

**Figure 14:**
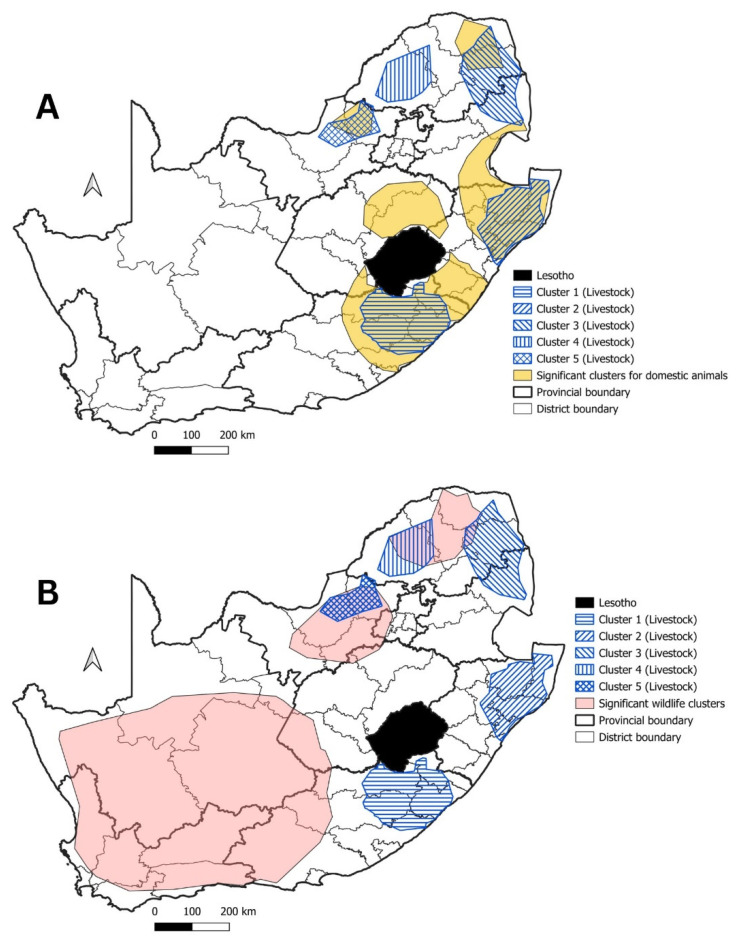
(**A**) Livestock clusters (indicated by the blue hashed lines) in relation to clusters in domestic animals; (**B**) significant disease clusters for livestock (indicated by the blue hashed lines) in relation to wildlife clusters.

To determine whether domestic animals or wildlife species were responsible for the livestock rabies cases, we compared the significant disease clusters for livestock with those observed for domestic animals and wildlife species. When comparing significant disease clusters between livestock and domestic animals, three of the livestock clusters in the EC, KZN, LP and NW provinces overlapped with significant disease clusters detected for domestic animal species (Figure 14A). Only one livestock disease cluster coincided with significant disease clusters observed for wildlife species in the LP province (Figure 14B). Additionally, only the livestock disease cluster in the NW province (Cluster 5) shared an overlap with significant disease clusters seen for both domestic animals and wildlife species.

## 4. Discussion

With rabies surveillance firmly rooted in both the implementation of disease intervention efforts, and the eventual self-declaration of freedom from canine-mediated rabies, it is vital that surveillance programs be thoroughly evaluated and improved where necessary. In this study, we utilized laboratory-derived surveillance data generated over a 21-year period for spatio-temporal scan statistics to identify significant disease clusters. This information was, in-turn, supplemented with disease testing rates and animal demographic data to gain insights into high-risk areas, and to the existing shortcomings of South Africa’s surveillance efforts.

Using the surveillance data generated between 1998 and 2019, we found that the majority of the samples that were sent for diagnostic testing in South Africa originated from the eastern half of the country (Figure 1). These observations aligned with the two studies that had also endeavored to gain an improved understanding of the epidemiology of rabies in South Africa [41,42]. As both laboratories responsible for animal rabies diagnosis in South Africa are also within the eastern half of the country where the majority of the samples had originated, we assessed the robustness of the rabies surveillance data used in this study. To this end, the AAHR for all the districts in the country was calculated and found that 26 (50%) of the country’s 52 districts had a AAHR value that was below the required threshold of 1.9 (Figure 2). Considering the diverse distribution of rabies reservoir species across South Africa, it was pertinent to investigate the surveillance data—disaggregated by the different species groups—in more detail.

In the case of domestic animals (which included domestic dogs and cats) in South Africa, the spatio-temporal analysis identified five significant rabies clusters (Figure 4). Three of these significant clusters (Cluster 1–3) were similar to the main focal source areas of canine rabies identified in the ‘National Strategy for the Elimination of Canine Mediated Human Rabies in South Africa (2019–2030)’ [31]. Furthermore, to evaluate where most of the human rabies cases occurred, the NICD recorded a total of 105 positive human rabies cases between 2008 and 2018. Most of these human rabies exposures and subsequent deaths occurred in the EC (*n* = 34), KZN (*n* = 31), and the LP (*n* = 22) provinces [40]. These provinces also accounted for most of the human rabies cases between 1997 and 2007, and the highest number of cases correlated to regions where significant canine rabies disease clusters could be detected. The results presented here also identified two significant disease clusters (Cluster 4 and 5) that were not indicated in the main focal source areas of canine rabies in South Africa’s National Strategic Plan (Figure 4). These clusters both had a shorter duration (both lasting approximately two years), and it could thus be speculated that they were associated with short-term rabies outbreaks that were geographically limited to the FS (Cluster 4) and NW (Cluster 5) provinces in 2014 and 2010, respectively.

To determine whether the absence of significant disease clusters in domestic animals in other geographical areas of the country were due to a true absence of risk or due to limited surveillance, we utilized the DAHR value for surveillance data collected between 2010 and 2019. Using this approach, 21 districts across South Africa were identified where the DAHR value was below the required threshold value of 0.8, indicating limited surveillance of domestic animals in the affected districts (Figure 5A). Of these 21 districts, only one district in the WC province (Figure 6) had an estimated dog population density that was theoretically too low to maintain rabies transmission. As a result, 20 at-risk districts (spread across the LP, NW, GP, NC, EC and WC provinces) were identified where surveillance of domestic animals was seemingly limited. Of the 20 identified districts, 8 (40%) were within or next to the significant disease clusters observed for domestic animals and 9 (17%) districts were within the WC and GP provinces that are not considered endemic for canine-mediated rabies (Figure 5B). Given the inadequate surveillance indicated by the testing rate analyses, the observed significant disease clusters are most likely smaller or less numerous than what they should have been. Increasing the surveillance capacity, therefore, might lead to the detection of larger disease clusters encompassing larger geographic regions.

In support of this notion, a rabies outbreak of unprecedented scale occurred in the Nelson Mandela Bay Metropolitan district of the EC province (one of the districts with inadequate rabies surveillance identified in this investigation) in 2022 [49]. In that investigation, the authors speculated that the outbreak could have been a continuation of the evolving epidemic of rabies in the eastern EC and KZN provinces (identified as Cluster 2 in this investigation) [49]. The impact of the EC disease outbreak on the size of the significant disease cluster that we identified in this investigation (Cluster 2) could not be determined as the surveillance data generated during the outbreak (2021 onwards) was not included in our dataset (1998 to 2019).

With a specific focus on wildlife rabies in South Africa, the spatio-temporal analysis identified three significant disease clusters associated with wildlife species other than mongooses (Figure 9). The observed significant disease clusters aligned with the findings of previous studies which suggested that high-density jackal populations in South Africa are mostly associated with rural farming regions and appear to be the dominant maintenance host in the northern areas of South Africa [14,16,22]. Meanwhile, despite having a relatively large geographical distribution throughout South Africa, bat-eared fox populations seem to be the dominant maintenance host in the western areas of the country [22] while an outbreak of rabies in aardwolf was reported in the NC province of South Africa in geographic locations overlapping with the known distribution of bat-eared foxes. It was noted that the number of positive rabies cases in aardwolf between 2011 and 2016 exceeded those seen in bat-eared foxes [24]. In contrast, rabies cases in mongoose populations (capable of transmitting the mongoose variant of the RABV) are endemic to the central plateau of South Africa, although the geographic distribution of various mongoose species overlap throughout the country [19,20,21].

With regard to the clusters observed for the non-mongoose wildlife species, Clusters 1 and 3 were both observed within the LP and NW provinces where black-backed jackals are known to be the primary sylvatic reservoir species of RABV [50] (Figure 10). Both of these clusters also overlapped to some extent with the significant disease clusters observed for domestic animals (Figure 5), suggesting potential virus spill-over between co-habiting wildlife species and unvaccinated domestic dogs [18,19,50]. In support of this finding, the interface between sylvatic and domestic dog species had previously been investigated in both the LP [50] and NW provinces [16]. In the first study, the authors found that blacked-backed jackals in the LP province were known to encounter free-roaming dogs in the villages adjacent to farming areas, allowing for continuous interaction between affected individuals [18]. In the second study, the authors provided strong evidence supporting cycles of both canine and wildlife rabies spread throughout the province [16]. Interestingly, the timeframe and geographical distribution of Cluster 3 in the NW province (2016–2019) coincided with an outbreak of rabies in black-backed jackals along the western boundary of the GP province after the disease expanded from the NW province in 2016 [17]. The third and final significant disease cluster in wildlife species other than mongooses (Cluster 2) was of considerable size and was seen where the home ranges of both bat-eared foxes and aardwolf species overlap (Figure 9 and Figure 10). The size of this cluster could be explained by the transient home ranges of bat-eared foxes and aardwolf that are known to constantly move around depending on rainfall and food sources, allowing them to disperse over greater areas and interact with other vulnerable species [51,52,53,54]. This would, in theory, allow for the maintenance of rabies within and between these species and allow for a larger significant disease cluster. It should, however, be noted that the size and disaggregation of the observed cluster is likely to have been influenced by the necessity to exclude several relevant records (*n* = 2019) from the spatio-temporal analysis on the basis that they lacked precise geographical location data.

While still focusing on wildlife rabies in the country, the spatio-temporal analysis further identified two significant disease clusters related to mongooses specifically (Figure 9). These two significant disease clusters were both geographically defined to relatively small regions of the FS and MP provinces, respectively. The observation of a significant mongoose rabies cluster in the FS province was not surprising, given historical evidence from this region of South Africa [21,55]. The second cluster, located in the MP province was restricted to a limited geographical range with the outbreak seemingly coming to an end in 2005. Between 1998 and 2007, mongoose rabies cases in the MP province were spread throughout the eastern and central parts of the province [14], with our spatio-temporal analysis identifying a significant disease cluster within that same geographical area (Figure 9). While this would explain the presence of the significant disease cluster, it seemingly spanned over two districts in the MP province. One of those districts had a WHR testing rate that was considerably lower than the districts surrounding the observed cluster. This would suggest that increased surveillance in that district could have resulted in the observation of a larger cluster—potentially spanning over a longer period of time—or additional rabies clusters forming over larger geographic areas.

Considering the widespread distribution of sylvatic reservoir species in South Africa (Figure 10), the limited geographical distribution of almost all the significant disease clusters in mongooses and other sylvatic species (except for Cluster 2 for the wildlife species) came as a surprise. The WHR values provided valuable insight by demonstrating that rabies surveillance in South Africa is seemingly biased towards domestic animals. The limitations associated with rabies surveillance in wildlife species had been investigated elsewhere where the authors found that wildlife rabies surveillance could be limited due to various factors, viz. bias as a result of personal interest [56,57], inconsistent surveillance [58,59] and low reporting rates [44,58,60]. In this investigation specifically, the WHR values were seemingly low in 29 of the districts that fell within the natural home ranges of the sylvatic reservoir species (Figure 11A). As such, had the WHR values been higher in those districts, the geographical range of the significant disease clusters observed for mongooses and other sylvatic species could have been broader.

Lastly, significant rabies disease clusters in livestock species in South Africa was evaluated. As livestock species are considered a dead-end host for rabies, rabies infection in livestock animals are either as a result from interactions with domestic dogs or wildlife species [50,61]. It was thus not surprising that the highest numbers of rabies cases in livestock originated from the eastern half of the country where rabies cases in domestic dogs were most prevalent. Of the significant disease clusters observed for livestock, three could be associated with clusters seen for domestic animals (Figure 14). To support the relationship between livestock and domestic dog rabies cases and outbreaks, two of the overlapping clusters for both domestic animals and livestock (Clusters 2 and 3) were detected in the same time periods (from 2000 to 2010 and from 2005 to 2015), respectively. The remaining livestock cluster in the EC province (Cluster 1) did overlap with a significant disease cluster for domestic animals, but the two clusters were not detected for the same time periods. This did not exclude domestic dogs from being the cause of the livestock cases as the surveillance in the EC province was known to be limited in all the testing rates we calculated.

Both wildlife species and domestic dogs had overlapping disease clusters with Cluster 5 (Figure 14A,B), indicating that both domestic dogs and wildlife may have been involved with the detection of a significant disease cluster in livestock in the NW province. These findings had previously been supported through a molecular epidemiological analysis where positive rabies samples from livestock could phylogenetically be linked to domestic dogs and an independent cycle of rabies in black-backed jackals [16].

The last remaining livestock cluster (Cluster 4) in the LP province overlapped with a wildlife disease cluster that occurred during the same time period. This would suggest that the livestock cluster in the LP province was caused by wildlife species, most notably black-backed jackals. This is not surprising as black-backed jackals in western LP have been shown to maintain rabies independently from domestic dogs and also coincides with geographic localities where farming activities are abundant [17,32,50].

## 5. Conclusions

A national passive rabies surveillance network has been established in South Africa for many years, but prior to this investigation, the robustness of this surveillance had not been fully assessed for its successes, or for the detection of potential limitations and shortcomings. By using spatio-temporal scan statistics, we have detected high-risk districts where disease intervention efforts would have the greatest impact on reducing the incidence of rabies in the country. However, by supplementing the outputs of the spatio-temporal scan statistics with rabies testing rates for different host species groups and the geographical distribution of these species, we also identified shortcomings associated with rabies surveillance in domestic animals that are located in specific districts of the country’s rabies-endemic provinces and the overall surveillance of wildlife species that are capable of maintaining rabies. By providing the resources required to increase rabies surveillance in the districts identified in this investigation, the high-risk areas for rabies could be accurately defined. In conclusion, the approach presented here allowed for the identification of areas of significant risk and underreporting and in the face of poor surveillance data in most of the rabies-endemic world, we argue that the wider application of the modified spatio-temporal scan statistical approach reported here, could significantly contribute to better control strategies in commitment to the elimination of dog-mediated rabies on a global scale.

## Figures and Tables

**Table 1:** SaTScan cluster information for each significant disease cluster observed for domestic animals.

Cluster	Start Date	End Date	*p*-Value	Observed Cases	Expected Cases	Relative Risk
1	1999	2009	*p* < 0.001	1455	835.0	1.94
2	2000	2010	*p* < 0.001	935	488.2	2.06
3	2005	2015	*p* < 0.001	447	231.8	1.99
4	2014	2016	*p* < 0.05	35	15.8	2.22
5	2010	2012	*p* < 0.05	96	58.6	1.65

**Table 2:** SaTScan cluster information for each cluster observed for wildlife and mongoose species.

Cluster	Species	Start Date	End Date	*p*-Value	Observed Cases	Expected Cases	Relative Risk
1	Wildlife	1998	2008	*p* < 0.001	178	74.8	2.67
2	Wildlife	1998	2008	*p* < 0.001	209	97.6	2.44
3	Wildlife	2016	2019	*p* < 0.001	87	37.2	2.46
1	Mongoose	1998	2008	*p* < 0.001	309	187.7	1.90
2	Mongoose	1998	2005	*p* < 0.05	77	43.8	1.82

**Table 3:** SaTScan cluster information for each cluster observed for livestock.

Cluster	Start Date	End Date	Log-Likelihood Ratio (*p*-Value)	Observed Cases	Expected Cases	Relative Risk
1	2002	2012	182.7 (*p* < 0.001)	410	210.1	2.13
2	2000	2009	42.0 (*p* < 0.001)	195	120.0	1.68
3	2005	2015	23.1 (*p* < 0.001)	133	85.6	1.58
4	2000	2003	14.3 (*p* < 0.05)	61	36.5	1.69
5	2014	2015	13.1 (*p* < 0.05)	44	24.7	1.79

## Data Availability

Surveillance data on rabies cases that support the findings of this study are available upon request from the Agricultural Research Council—Onderstepoort Veterinary Research, Pretoria, South Africa (https://www.arc.agric.za/arc-ovi/Pages/ARC-OVI-Homepage.aspx (accessed on 28 March 2024)). The data cannot be shared publicly due to ethical restrictions. The original contributions presented (through figures and tables) in this study are included in this article/Supplementary Materials, further inquiries can be directed to the corresponding author. The raw analysis data not included as Supplementary Materials supporting the conclusions of this article will be made available by the authors upon request.

## Supplementary Materials

The following supporting information can be downloaded at: https://www.mdpi.com/article/10.3390/tropicalmed9060122/s1, Table S1: Total number of rabies cases per species group across South Africa between 1998 and 2019; Table S2: Total positive and negative cases per species group in each province of South Africa between 1998 and 2019.
