# Supplementary material for: A Perspective of the Epidemiology of Rabies in South Africa, 1998–2019
Source: Trop Med Infect Dis. 2024 May 22;9(6):122. doi: 10.3390/tropicalmed9060122 (PMC11209162; doi:10.3390/tropicalmed9060122)
Supplement: Supplementary file 1 [file tropicalmed-09-00122-s001.zip › tropicalmed-3003010-supplementary.pdf]

Table S1: Total number of rabies cases per species group across South Africa between 1998 and 2019.

Species group	Positive cases	Negative cases	% Positive
**Dog**	6,682	12,254	35.3
**Livestock**	2,645	3,696	41.7
**Mongoose**	1,107	2,683	29.2
**Wildlife**	1,141	3,436	24.9
**Cat**	332	3,063	9.78
**Total**	**11,907**	**25,132**	**32.1**

Table S2: Total positive and negative cases per species group in each province of South Africa between 1998 and 2019.

Province	Result	Canine	Feline	Livestock	Mongoose	Wildlife
Eastern Cape	Positive	877	32	857	68	64
	Negative	857	161	446	178	249
Free State	Positive	408	137	407	623	125
	Negative	950	427	859	883	540
Gauteng	Positive	74	5	27	25	41
	Negative	1048	370	237	293	315
KwaZulu-Natal	Positive	3601	58	537	34	69
	Negative	6274	1337	745	220	668
Limpopo	Positive	699	10	332	8	283
	Negative	630	114	418	101	336
Mpumalanga	Positive	911	16	223	163	24
	Negative	1713	261	388	426	569
North West	Positive	108	26	238	108	109
	Negative	371	178	434	279	273
Northern Cape	Positive	32	38	27	63	286
	Negative	221	106	115	175	289
Western Cape	Positive	6	11	5	15	155
	Negative	247	121	72	131	223
